# Effects of Essential Oil Inhalation on the Enhancement of Plasma and Liver Lipid Metabolism in Mice

**DOI:** 10.3390/ijms26125674

**Published:** 2025-06-13

**Authors:** Junko Shibato, Ai Kimura, Michio Yamashita, Seiji Shioda, Fumiko Takenoya, Randeep Rakwal

**Affiliations:** 1 Department of Functional Morphology, Shonan University of Medical Sciences, Yokohama 244-0806, Japan; rjunko@nifty.com (J.S.); seiji.shioda@sums.ac.jp (S.S.); 2 Department of Sport Sciences, School of Pharmacy and Pharmaceutical Sciences, Hoshi University, Tokyo 142-8501, Japan; francfranc.fragola@gmail.com (A.K.); d1902@hoshi.ac.jp (M.Y.); 3 Institute of Health and Sport Sciences, Tsukuba International Academy for Sport Studies (TIAS2.0), University of Tsukuba, Tsukuba 305-8574, Japan

**Keywords:** aromatherapy, essential oil inhalation, *Citrus aurantium*, *Lavandula angustifolia* gene expression, lipid metabolism, anti-obesity

## Abstract

The purpose of this study was to determine the effects of essential oil inhalation on body weight, blood lipid profile, and liver and adipose tissue in mice. Middle-aged male mice (C57BL/6J) were exposed to *Lavandula angustifolia* (LO) and *Citrus aurantium* (CAO) essential oils for 7 weeks and compared to mice that did not receive essential oil inhalation treatment. Liver, white adipose tissue, and brown adipose tissue were sampled, kept at −80 °C. Although essential oil inhalation increased feed intake and body weight compared to control group, the amount of weight gain per feed intake was lower in the *C. aurantium* essential oil group. Moreover, relative weight of fat to body weight, liver fat amount, and blood cholesterol was lower, and triglyceride levels were significantly reduced. Reverse transcription polymerase chain reaction (RT-PCR) expression profiling of genes related to lipid metabolism confirmed changes in the regulation of thermogenesis-related gene *Ucp1* and the cholesterol synthesis-related genes *Hmgcs1* and *Hmgcr*. The inhalation of *C. aurantium* essential oil did not reduce the feed intake in mice; however, its effectiveness in suppressing the increases in body weight and fat mass was demonstrated.

## 1. Introduction

The effects of essential oils through aromatherapy are used as a complementary therapy to promote and maintain human psychological and physiological health. Essential oils are administered by oral, dermal, and intranasal means. Although most of the uses of essential oils are in the field of cosmetics and perfumes, essential oils have been known since ancient times to have bactericidal and antibacterial properties and have been widely used as antiseptics and infection preventatives. Furthermore, essential oils have anti-inflammatory and antioxidant activity, suggesting the possibility of dietary essential oil supplementation in the prevention of several diseases, including brain dysfunction, cancer, heart disease, and impaired immunity.

The physiological and pharmacological effects of aromatherapy using essential oils have been shown to include antibacterial, anti-stress, anti-depressant, anti-inflammatory, antioxidant, and relaxing effects [1]. The oil is volatile, easily crosses the blood–brain barrier, and also acts directly on brain neurons [2]. Essential oil odor exposure has demonstrated neuropharmacological effects in animal models by affecting multiple neurotransmitter systems [3]. However, much of the research on the pharmacological effects of essential oils due to their olfactory stimulation has focused on their relaxing effects. In other words, essential oils are promoted for their anti-anxiety effects [4,5].

*Citrus aurantium* (bitter orange) is native to East Asia and is widely cultivated in the warmer regions of the planet such as the Mediterranean, African continent, South America, and California. The bitter orange essential oil obtained by pressing the peel is used in the food industry, especially in liqueurs and soft drinks. It is widely used as a fragrance. Bitter orange essential oil is extracted from the rind, but sweet orange, neroli for flowers, and petitgrain for leaves are produced from the fruit [6] and are widely used in aromatherapy for relaxation, sleep promotion, and digestion. Inhalation of *C. aurantium* essential oil has been reported to have sedative and relaxing effects in rats and mice [7,8,9], to relieve anxiety and pain specific to women (labor and delivery) [10,11], mild to moderate pain relief after orthopedic surgery [12], and to improve sleep quality [13,14].

Recent studies have demonstrated that essential oils and aromatic compounds regulate food intake and energy expenditure. Patchouli essential oil [15], citronella oil [16], fennel essential oil [17], and grapefruit oil [18] have all been shown to reduce food intake and body weight and improve lipid metabolism by inhalation. On the other hand, olfactory stimulation from the smell of lavender essential oil suppressed lipolysis and increased appetite and weight [19]. These reports suggest that the scent of essential oils can be used to help people lose weight in obese people and stimulate appetite in people with anorexia, and that aromatherapy can contribute to the prevention and treatment of eating disorders.

The active ingredient synephrine, which is abundant in the extract of the immature fruit of *C. aurantium*, has been reported in studies to act on fat cells, promotes the burning of body fat [20,21,22] and is used as a dietary supplement [23,24], and is widely used as a sports performance enhancer [25,26,27]. However, oral administration has been the most common method of administration in these studies. The National Collegiate Athletic Association (NCAA) has listed synephrine (bitter orange) as a stimulant on its most recent list of banned substances [28]. Although synephrine is not currently a prohibited substance, it is listed as a stimulant in the World Anti-Doping Code Monitoring Program, the international standard for the 2025 Prohibited List.

Improving lipid metabolism by inhalation, which is safer, is therefore attractive. However, there are fewer studies showing that essential oil inhalation acts on weight loss and heat production in adipose tissue. The present study therefore aimed to determine the potential of aromatherapy to improve lipid metabolism by investigating the effects of inhalation of *C. aurantium* peel essential oil (CAO) and *Lavandula angustifolia* flower bud essential oil (LO) on body weight, blood lipid profile, and liver and adipose tissue in middle-aged mice.

## 2. Results

### 2.1. Effects of Essential Oil Inhalation on Mouse (C57BL/6J) Food Intake, Body Weight, and Fat Mass

**Figure 1:**
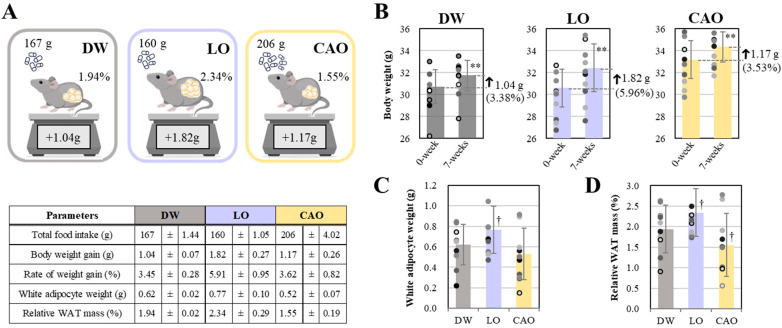
Total food intake (g), body weight gain (g), and relative white adipocyte tissue (WAT) mass (%) (**A**) of mice (C57BL/6J) inhaling essential oils for 7 weeks. Body weight gain (g) and percentage gain (%) (**B**) of mice after 7 weeks of inhalation of essential oils compared to body weight at the start of the experiment. White adipocyte weight (g) and relative WAT mass (%) (**C**,**D**) of mice after inhalation of essential oils for 7 weeks. DW (distilled water), LO (*Lavandula angustifolia)*, and CAO (*Citrus aurantium)* are shown as results for groups of 10 mice (*n* = 10). Error bars represent SE (mean ± standard error). Significant: ** *p* < 0.01 vs. DW; significant trend: † 0.05 ≤ *p* < 0.1 vs DW; Tukey method (*n* = 10).

Total food intake (g), body weight gain (g), white fat cell mass (g), and relative fat organ mass (%) of mice (C57BL/6J) after 7 weeks of essential oil (*Lavandula angustifolia*) (*Citrus aurantium*) inhalation are shown in Figure 1. As shown in Figure 1A (next page), the total food intake for 7 weeks was 167 g (DW, distilled water), 160 g (LO, *L. angustifolia* flower bud essential oil), and 206 g (CAO, *C. aurantium* peel essential oil). And, as shown in Figure 1B, the weight gain and percentage gain of mice after 7 weeks of essential oil inhalation was 1.04 g (3.38%, *p* value = 0.003) for DW, 1.82 g (5.96%, *p* value = 4 × 10^−6^) for LO, and 1.17 g (3.53%, *p* value = 0.001) for CAO. The increase in white fat cell mass (g) of mice after 7 weeks of inhalation showed a significant trend for LO compared with DW (*p* value = 0.08) (Figure 1C). Furthermore, relative fat organ mass (%) showed a significant trend for an increase in LO (*p* value = 0.078) and a decrease in CAO compared to DW (*p* value = 0.095) (Figure 1D).

Although there was statistical difference, the data under the present conditions show that LO inhalation mice had a lower total food intake. However, a tendency of increase in the rate of weight gain and relative fat organ mass was seen. Similarly, in the case of CAO inhalation, mice had higher total food intake. Additionally, a tendency to inhibit the rate of weight gain and a decrease in relative fat organ mass was observed.

### 2.2. Effect of Essential Oil Inhalation on Liver Lipid Accumulation in Mice

**Figure 2:**
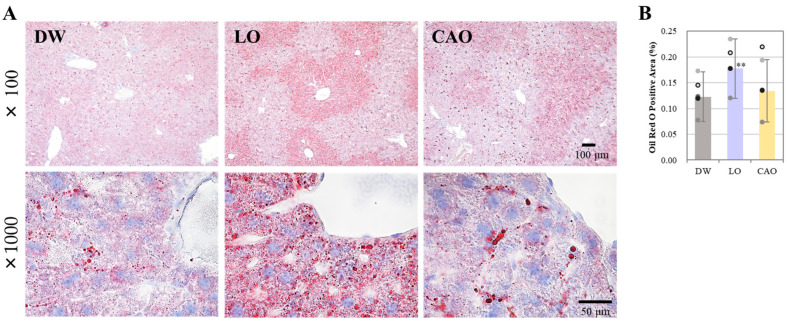
Testing the effect of essential oil inhalation on hepatic lipid accumulation in mice (C57BL/6J). Liver sections from the DW (distilled water), LO (*Lavandula angustifolia*), and CAO (*Citrus aurantium*) groups were stained with Oil Red O. (**A**) 100× magnification, scale bar 100 µm, and 1000× magnification, scale bar 50 µm. Quantification of Oil Red O staining was performed using imageJ (**B**). Error bars represent SE (mean ± standard error). ** *p* < 0.01 vs. DW, Tukey method (*n* = 5).

To examine the effect of essential oil inhalation on liver lipid accumulation, liver sections from the DW, LO, and CAO groups were stained with Oil Red O. The results are shown in Figure 2A (next page), and a graphical representation of the Oil Red O staining area can be seen in Figure 2B. At 7 weeks after essential oil inhalation, liver lipid accumulation was significantly increased in LO compared with DW (*p* value = 0.001). CAO showed more lipid accumulation in staining compared to DW, but no significant difference was identified.

### 2.3. Effect of Essential Oil Inhalation on Blood Lipid Profiles of Mice

**Figure 3:**
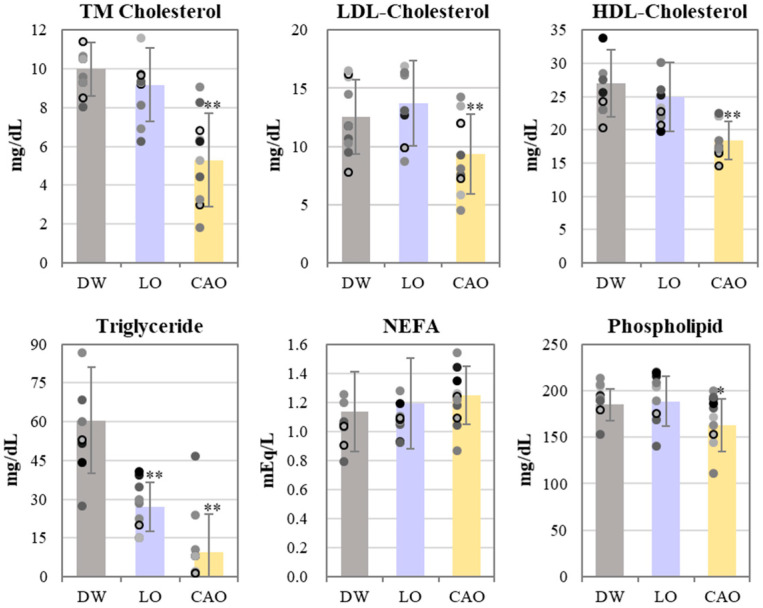
The blood lipid profile of mice (C57BL/6J) inhaling essential oil for 7 weeks. Values of total cholesterol, LDL cholesterol, HDL cholesterol, triglycerides, free fatty acids and phospholipids in blood absorbing DW (distilled water), LO (*Lavandula angustifolia)*, and CAO (*Citrus aurantium)* for 7 weeks. Error bars represent SE (mean ± standard error). ** *p* < 0.01; * *p* < 0.05 vs. DW; Tukey method (*n* = 10).

The results of the blood lipid profile of mice inhaling essential oil for 7 weeks are shown in Figure 3. Total cholesterol (TM Cholesterol), LDL cholesterol, and HDL cholesterol were significantly reduced in CAO compared to DW (LDL: *p* value = 0.014, HDL: *p* value = 0.002). Triglycerides were significantly reduced in LO and CAO (LO: *p* value = 7 × 10^−4^, CAO: *p* value = 3 × 10^−5^), but CAO was found to be more reduced compared to LO; NEFAs (free fatty acids) were unchanged and phospholipids were found to be significantly reduced in CAO (*p* value = 0.051).

### 2.4. Effects of Essential Oil Inhalation on Genes Associated with Adipogenesis

**Figure 4:**
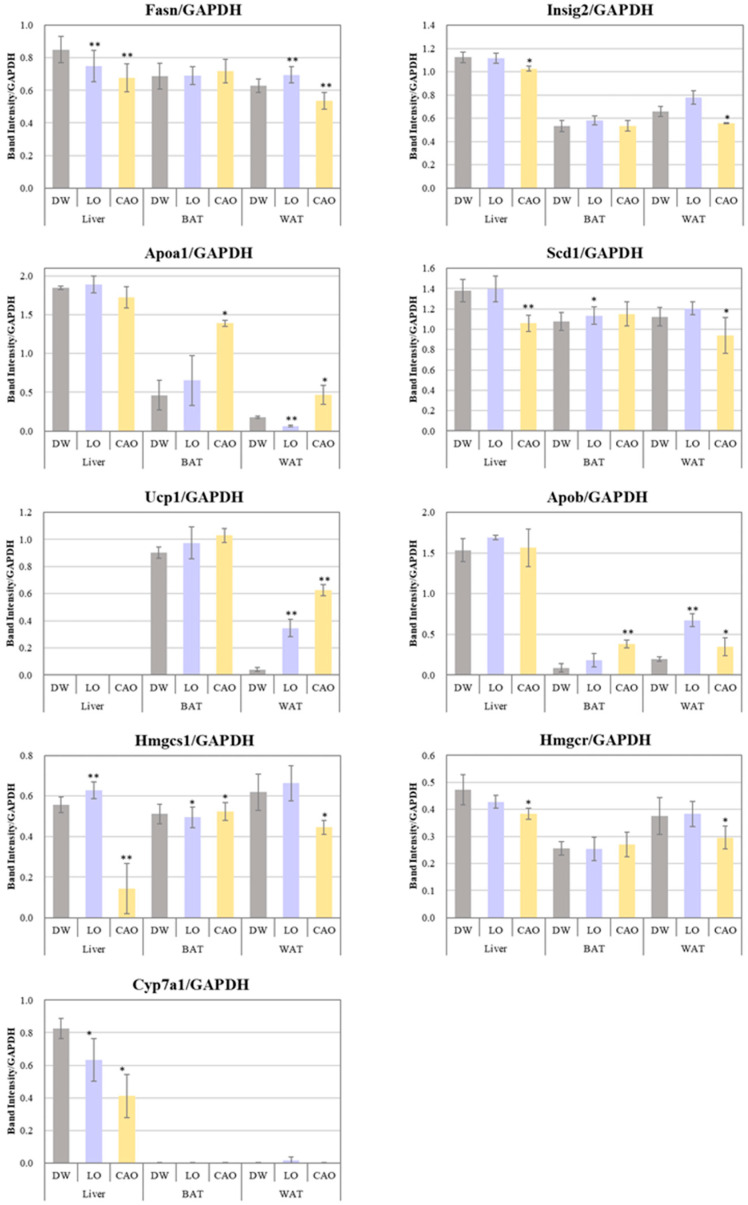
Effect of 7 weeks of essential oil inhalation on gene expression in the mice (C57BL/6J) liver, WAT (white adipose tissue), and BAT (brown adipose tissue). Band intensities of RT-PCR products after electrophoresis were corrected for *GAPDH* and are displayed graphically. Error bars represent SE (mean ± standard error). ** *p* < 0.01; * *p* < 0.05 vs. DW (distilled water); Tukey method (mouse tissue—*n* = 10; PCR technical replicate—*n* = 3).

The mRNA expression levels of adipogenesis genes (*Fasn*, *Scd1*, *Insig2*), LDL transport protein genes (*Apob*), HDL biosynthesis genes (*Apoa1*), fat burning (*Ucp1*), cholesterol biosynthesis genes (*Hmgcr*, *Hmgcs1*), and bile acid biosynthesis (*Cyp7a1*) were assessed in WAT, BAT, and liver to determine whether essential oil inhalation affects the expression of genes associated with adipogenesis (Figure 4).

Compared to DW, the *Fasn*, *Scd1*, *Insig2*, *Hmgcr*, *Hmgcs1*, and *Cyp7a1* gene expression was significantly down-regulated in CAO compared to DW in liver (*p* value = *Fasn*: 0.005; *Scd1*: 0.004; *Insig2*: 0.036; *Hmgcr*: 0.018; *Hmgcs1*: 0.007; *Cyp7a1*: 0.014) and WAT (*p* value = *Fasn*: 0.009; *Scd1*: 0.026; *Insig2*: 0.016; *Hmgcr*: 0.042), and the *Apob*, *Apoa1*, and *Ucp1* genes were significantly up-regulated in BAT (*p* value = *Apob*: 0.005; *Apoa1*: 0.012) and WAT (*p* value = *Apob*: 0.058; *Apoa1*: 0.034; *Ucp1*: 0.001). On the other hand, compared to DW, *Fasn* and *Cyp7a1* in LO were significantly down-regulated in liver (*p* value = *Fasn*: 0.006; *Cyp7a1*: 0.042) and WAT (*p* value = *Fasn*: 0.004), as well as the *Apoa1* in WAT (*p* value = *Apoa1*: 3 × 10^−4^) and *Hmgcs1* genes in BAT (*Hmgcs1*: 0.024). Furthermore, compared to DW, the *Fasn*, *Apob*, and *Ucp1* in LO WAT (*p* value = *Apob*: 0.009, *Ucp1*: 0.006) and *Scd1* (*p* value = 0.038) in BAT were significantly up-regulated.

## 3. Discussion

**Figure 5:**
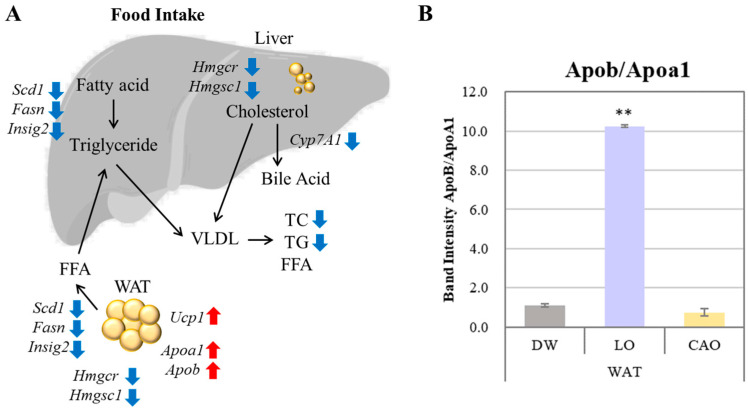
Gene expression changes in mouse (C57BL/6J) liver and WAT (white adipose tissue) after 7 weeks of CAO (*C. aurantium*) inhalation (**A**); *Apob*/*Apoa1* gene expression ratio in WAT after 7 weeks of essential oil inhalation (**B**); Error bars represent SE (mean ± standard error). Tukey method (mouse tissue *n* = 10, PCR technical replicate *n* = 3). VLDL: very low density lipoprotein; TC: total cholesterol; TG: triglyceride; FFA: free fatty acids. ** *p* < 0.01. Increased expression is indicated by red arrows and decreased expression by blue arrows.

Inhalation of essential oil from the peel of *C. aurantium* was shown to inhibit weight gain despite the increased food intake (no statistical difference). Additionally, it also lowered the serum cholesterol levels. While it is a natural consequence of reduced food intake that both weight gain and serum cholesterol level decrease, the data from the present study shows that CAO inhalation inhibits both weight and fat gain without reducing food intake (no statistical difference). And it also lowers serum cholesterol and triglyceride levels. The effect of CAO on weight gain can be explained by the decreased expression of the liposynthesis genes *Fasn*, *Scd1*, and *Insig2* and the cholesterol synthesis genes *Hmgcr* and *Hmgcs1* in the liver and WAT, as well as the fat burning effect due to increased expression of the *Ucp1* gene in the WAT (Figure 5A). In contrast, LO showed an increased expression of *Ucp1* genes in WAT, but no suppression in the case of the *Fasn*, *Scd1*, *Insig2*, *Hmgcr*, and *Hmgcs1* genes. Hmgcs1 in particular is an important enzyme in the mevalonic acid pathway of cholesterol synthesis. Recent studies have shown that dyslipidemia can be treated by either reducing the expression of *Hmgcs1* or directly inhibiting its activity [25,26]. Statins used as standard treatment for patients with high cholesterol are Hmgcs1 inhibitors. Therefore, the increased *Hmgcs1* expression in the liver and WAT with LO inhalation compared to DW, and decreased *Hmgcs1* expression with CAO inhalation, was correlated with the results of increased relative fat organ mass (%) and liver fat mass with LO, which decreased with CAO (Figure 5A).

*Apoa1* and *Apob* gene expression was up-regulated in adipose tissue by LO and CAO essential oil inhalation (Figure 5A). ApoA1 constitutes the major protein component of HDL and promotes the reverse transport of cholesterol from peripheral tissues to the liver, exerting an anti-arteriosclerotic effect. ApoB is a major protein component of triglyceride-rich lipoproteins and is involved in the transport of cholesterol from liver cells to peripheral cells, promoting cholesterol deposition in arteries and is a known indicator of acute myocardial infarction risk. The results of *Apoa1* and *Apob* gene expression in WAT by inhalation of LO and CAO essential oil in the present study are shown graphically as the *ApoB/ApoA1* ratio (Figure 5B). The results showed a higher *ApoB/ApoA1* ratio in LO compared to DW and CAO; the *ApoB/ApoA1* ratio is considered a strong predictor of atherosclerotic cardiovascular disease [29]. Therefore, although *Apoa1* and *Apob* gene expression was enhanced by CAO essential oil inhalation, the *ApoB/ApoA1* ratio was not high, which may be related to the lower relative fat organ mass (%) in CAO than in LO.

Cyp7a1 (cholesterol 7α-hydroxylase) is the rate-limiting enzyme that regulates the biosynthesis of bile acids. Many studies have reported that activating Cyp7a1 to promote bile acid synthesis can reduce high-fat diet-induced hypercholesterolemia [27]. In the present study, CAO was found to down-regulate the *Cyp7a1* gene, which may be partly due to the fact that all reported studies of the anti-obesity effects of Cyp7a1 activation are under conditions of a high-fat diet rather than a normal diet. Increased cholesterol in the liver increases *Cyp7a1* expression, which increases cholesterol extravasation [28,30,31], and since Cyp7a1 promotes bile acid synthesis reactions using cholesterol as a substrate, the down-regulation of *Cyp7a1* by CAO inhalation in the present study may be a possible mechanism to increase hepatic lipid and cholesterol synthesis-related genes *Fasn*, *Scd1*, *Insig2*, *Hmgcr*, and *Hmgcs1*, suggesting that it is associated with the suppression of these genes.

About 96% of the components of CAO are the monoterpene hydrocarbon limonene. Inhalation of limonene has been reported to have anti-inflammatory [32,33], anti-stress [34], and analgesic effects [35] in addition to antioxidant effects, but there are no reports of improved lipid metabolism. β-myrcene, which has the next highest content (1.59%) after limonene, has been reported to have anxiolytic and sedative effects by inhalation [36], but like limonene, there are no reports on improving lipid metabolism. Therefore, in future studies, it would be desirable to conduct more detailed research on which components of CAOs improve lipid metabolism in order to elucidate the anti-obesity effects of CAO inhalation.

## 4. Materials and Methods

### 4.1. Mouse Maintenance and Essential Oil Inhalation

**Figure 6:**
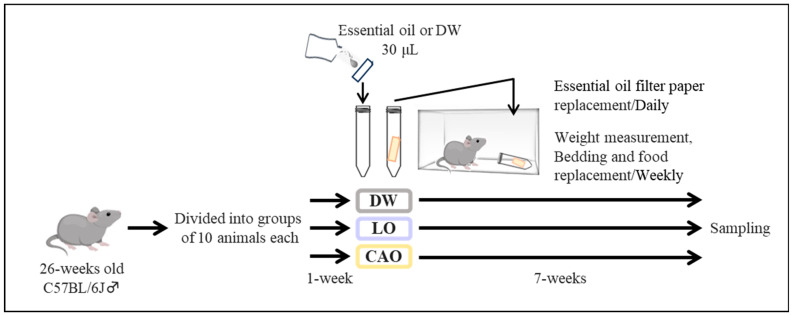
The study experimental design and essential oil exposure methodology for this research.

The essential oil inhalation method in mice is shown in Figure 6 (next page). Twenty-six-week-old male mice were acclimatized for one week, and twenty-seven-week-old mice were used for the exposure experiments. Briefly, the 27-week-old C57BL/6J male mice (purchased from Tokyo Laboratory Animals Science Co., Ltd. Tokyo, Japan) were randomly allocated to groups of 10 and exposed to each essential oil (LO (*Lavandula angustifolia*); CAO (*Citrus aurantium*)) or DW (distilled water) continuously on a daily basis after 1 week of acclimatization. Essential oil exposure was performed with 5 mice per cage. After 7 weeks, animals were weighed and dissected to obtain the samples for analyses (Figure 6).

The 26-week-old C57BL/6J male mice were randomly divided into groups of 10 each (n = 10) into DW (distilled water), LO (*Lavandula angustifolia*; P-98: PRANAROM), and CAO (*Citrus aurantium*; P-37: PRANAROM) for 1 week and acclimatized. The essential oils used were purchased as commercially available oils from PRANAROM (Hainaut, Belgium). The table of the compositional analysis (by GC-MS; Gas Chromatography–Mass Spectrometry) of PRANAROM′s essential Oils, CAO and LO, used in this study are presented in (Supplementary Data S1).

Mice were housed ad libitum at 22 ± 2 °C from 6:00 to 18:00 with a 12 h/12 h light/dark cycle. Solid feed for standard mouse rearing was purchased from Oriental Yeast Co., Ltd. (Tokyo, Japan). The ingredient list is shown in (Supplementary Data S2). After acclimation, 10 randomly assigned mice were continuously exposed to each essential oil (LO, CAO) or DW daily. Essential oil exposure was performed with 5 mice per cage. Essential oil exposure was performed in cages (5 mice/cage) using 1.5 mL microfuge tubes containing filter paper with 30 μL of essential oil. The tubes were left in the cage with the tube cap open and the filter paper containing the essential oil was changed daily at 10 a.m. The condition of 30 μL of essential oil was not evaluated this time, but no mice died or became sick due to the applied concentration. Therefore, it could be that it was not toxic to mice in the examination experiment. Body weight was measured weekly, and bedding and food were changed on the same day. After measuring body weight and diet at 7 weeks, the animals were dissected for sample collection. No animals were excluded from the sample/present study.

### 4.2. Sampling

All 10 mice exposed to each essential oil (LO, CAO) were used for sampling. Seven weeks after the start of essential oil inhalation, triple anesthesia (Medetomidine (Kyoritsu Seiyaku Corporation, Tokyo, Japan), Midazolam (Nichi-Iko Pharmaceutical Co., Ltd., Toyama, Japan), and Putorphanol (Meiji Seika Pharma Co., Ltd., Tokyo, Japan)) were administered, and cardiac blood was collected. Thereafter, the liver, white adipose tissue (WAT), and brown adipose tissue (BAT) were sampled. The collected blood was passed through blood heparin (Mochida Pharmaceutical Co., Ltd., Tokyo, Japan), centrifuged at 4 °C, 11,000 rpm for 10 min, and the supernatant was stored at −80 °C. A portion of the liver was saved for frozen sectioning. Liver, WAT, and BAT were stored at −80 °C until ribonucleic acid (RNA) extraction.

### 4.3. Oil Red O Staining

Five samples of liver used for Oil Red O were selected that were close to the average body weight after each essential oil (LO, CAO) exposure. Livers were fixed in 4% paraformaldehyde/PB solution overnight at 4 °C. Fixed tissues were replaced with PBS for 24 h, 20% sucrose for 24 h, 30% sucrose for 48 h, and frozen embedded. Frozen sections were prepared. Oil Red O (Muto Pure Chemicals Co., Ltd., Tokyo, Japan). Oil Red O was prepared by mixing Oil Red O and DW (Oil Red O/DW = 6:4) before use and filtered to obtain a staining solution. Frozen section slides were air-dried for 5 min and soaked in 60% isopropanol (Fujifilm Wako Pure Chemical Corporation, Osaka, Japan). The staining solution was added dropwise and allowed to stand at 37 °C for 15 min. The cells were quickly passed through 60% isopropanol, nuclear staining was performed with hematoxylin (Fujifilm Wako), and the cells were mounted. Section images were taken using a BZ-X 710 bright field system (Keyence Corp, Osaka, Japan), and images were analyzed using ImageJ software, version 1.54. (National Institute of Health, Bethesda, MD, USA).

### 4.4. LabAssay

Triglycerides, NEFAs (non-esterified fatty acids), cholesterol, LDL cholesterol, and high-density lipoprotein (HDL) cholesterol in the plasma of 10 mice exposed to each essential oil (LO, CAO) were detected using LabAssay (Fujifilm Wako), following the manufacturer’s protocol.

### 4.5. Total RNA Extraction for Gene Expression Analysis

Liver, WAT, and brown adipose tissue (*n* = 10) stored at −80 °C were ground in a mortar in liquid nitrogen, mixed with QIAzol Lysis Reagent (Qiagen, Hilden, Germany), and then processed using the RNeasy Mini Kit (Qiagen). Total RNA was extracted according to the manufacturer’s protocol. The RNA concentration of the extracted RNA was measured using a microvolume spectrophotometer (DS-11, DeNovix, Wilmington, DE, USA), and the 260/280 and 260/230 ratios were confirmed to be 1.8 or higher.

### 4.6. Reverse Transcription Polymerase Chain Reaction (RT-PCR)

The cDNA was synthesized from extracted RNA using Affinity Script QPCR cDNA synthesis kit (Agilent, Santa Clara, CA, USA). PCR reactions were performed using Emerald Amp PCR Master (Takara, Kusatsu, Japan) using primers specific to the gene of interest (Table 1). The PCR reaction consisted of initial denaturation at 97 °C for 5 min, heat denaturation at 95 °C for 45 s, annealing at 55 °C for 45 s, extension at 72 °C for 1 min, 27–36 cycles, and extension at 72 °C for 10 min. After the PCR reaction, the PCR products were separated on a 1.5% agarose gel and visualized with ethidium bromide under UV light [37]. The expression levels of the visualized target genes were corrected for the expression level of the GAPDH gene, a known housekeeping gene, and graphed with the average of three PCR reactions for each gene.

### 4.7. Statistical Analysis

Data are expressed as mean ± standard error (SE). Statistical comparisons were made using the Tukey test for multiple comparisons. *p*-values less than 0.05 were considered significant, and *p*-values between 0.05 and 0.1 were considered a significant trend. Significant: ** *p* < 0.01; * *p* < 0.05; significant trend: † 0.05 ≤ *p* < 0.1.

## 5. Conclusions

Aromatherapy may be a safe and efficient intervention and a simple and applicable tool for an actionable affect towards improving eating disorders and lipid metabolism. The identification of the beneficial effects of the diverse bioactive substances in citrus peels used for essential oils could also be aimed at valorizing waste products and improving the quality of by-products. In this study, CAO inhalation was found to improve lipid metabolism in the blood and liver. However, the mechanism underlying the increased food intake due to CAO and LO inhalation is not clear.

Our laboratory has been experimenting with essential oil exposure to regulate feeding in mice for quite a few years. Leite and co-workers [7] describe essential oil exposure methods and, like numerous references, examine many conditions such as dilution of essential oil, time, and high-fat diet. However, based on our (group) experience to date, it has been understood that most of the conditions do not yield clear results. So, we reviewed all the related literature, and combined with our experience (previous research), the experimental design of this study was prepared. Here, we use middle-aged mice maintained on a normal diet, and the essential oil was simply changed daily with 30 microliters of undiluted solution for 7 weeks; we do not believe there are, nor have we identified, any reports of experiments with a similar design.

It is known that the scent of essential oils affects physiological functions. It was confirmed that neurotransmission by the scent of osmanthus decreased the mRNA expression of appetite-promoting neuropeptides such as agouti-related protein, neuropeptide Y, melanin-concentrating hormone, and prepro-orexin, and further increased anorexia neuropeptides, which suppressed feeding [38]. Furthermore, our laboratory has confirmed that the scent of ginger essential oil may promote feeding via hypothalamic MCH [39]. However, further research is needed to understand the potential benefits of essential oil inhalation, including whether odor stimulation affects neuropeptide-containing neurons in the hypothalamus that control appetite.

There are few reports of experiments on lipid metabolism by inhalation of essential oil, and the purpose of this experiment was to obtain basic data. C57BL/6J mice, which have high genetic uniformity and good experimental reproducibility, are used as models for many human diseases, including obesity and diabetes. Thus, C57BL/6J mice were used for this study. Moreover, the experimental methods and conditions of this study are likely to generalize to other species. The essential oils used in this study are commonly used (e.g., at home), and nasal inhalation is a simple method. Also, the evaluation of blood samples has very high potential for use in human clinical experiments.

Although the effects of nasal inhalation of essential oil on lipid metabolism and body weight in mice were confirmed, the essential oil aromatic components that affect lipid metabolism and body weight remain to be determined. Based on the component list of CAO, limonene is considered to be highly involved in the fat metabolism effects observed in this study. Since limonene accounts for 95.72% of the total amount of the essential oil, there are reports of the anti-obesity effects of oral administration of limonene [40,41,42]. In the future, we would like to examine whether a similar effect can be obtained by intranasal administration of limonene alone. The experimental design presented in this study is characterized by its ease of application to humans. We would like to conduct the human clinical trials in the future to identify essential oil components that affect lipid metabolism and body weight, and to apply the results not only for weight loss, but also for the elderly and others who need to gain weight in a healthy manner.

## Figures and Tables

**Table 1:** The primer sequences and each primer set (gene-specific primers) used for the gene expression analyses experiment in this study.

	Forward Primer	Reverse Primer	
Accession	Nucleotide Sequence (5′-3′)	Nucleotide Sequence (5′-3′)	Gene Name
NM_007988	AATCCATCATCAACATCATCCA	CCACTGACTCTTCACAGACCAG	*Fasn*
NM_009692	ACGAATTCCAGAAGAAATGGAA	GTGGTACTCGTTCAAGGTAGGG	*Apoa1*
NM_009127	CACCTTCTTGCGATACACTCTG	CTCCCGTCTCCAGTTCTCTTAA	*Scd1*
NM_009463	AACTCTCTGCCAGGACAGTACC	AACGGAGCTGTTCATTTGATTT	*Ucp1*
NM_009693	GTTGGTGAGTCCACAAGATTGA	GCTTGGTTGCAGGTATAGTTCC	*Apob*
NM_145942	TGGTATCTGGTCAGAGTGGATG	GACCACAACAGGAAGCATGTTA	*Hmgcs1*
NM_001360166	TCACATGGTT CACAACAGAT CA	GCAC AGAGACTCCT CAGATGTG	*Hmgcr*
NM_153526	ACCACGTCTGGAACTATCCAAG	CTCCCAGGTGACTGTCAATACA	*Insig1*
NM_007824	AAATACGACCGGTACCTTGATG	TAACGCTCAGCAGTCGTTACAT	*Cyp7a1*
NM_001001303	GCTACACTGAGGACCAGGTTGT	CTCCTGTTATTATGGGGGTCTG	*GAPDH*

## Data Availability

The data presented in this study are available in the article. The raw data are available upon reasonable request from the corresponding author.

## Supplementary Materials

The following supporting information can be downloaded at: https://www.mdpi.com/article/10.3390/ijms26125674/s1.
