# Supplementary material for: Effects of Essential Oil Inhalation on the Enhancement of Plasma and Liver Lipid Metabolism in Mice
Source: Int J Mol Sci. 2025 Jun 13;26(12):5674. doi: 10.3390/ijms26125674 (PMC12192594; doi:10.3390/ijms26125674)
Supplement: Supplementary file 1 [file ijms-26-05674-s001.zip › ijms-3664767-supplementary.pdf]

Supplementary Data 1. Table of the compositional analysis of PRANAROM's essential Oils, CAO and LO used in this study. See, 4. Materials and Methods, section 4.1.

CAO		*Citrus aurantium* P-37: PRANAROM	
モノテルペン炭化水素	96.46%	Monoterpene Hydrocarbons	96.46%
α-Pinene	0.37%	α-Pinene	0.37%
β-Pinene	0.10%	β-Pinene	0.10%
β-ミルセン	1.74%	β-Myrcene	1.74%
リモネン	93.83%	Limonene	93.83%
β-フェランドレン	0.18%	β-Phellandrene	0.18%
trans-β-オシメン	0.16%	trans-β-Ocimene	0.16%
p-サイメン	0.08%	p-Symene	0.08%
モノテルペンアルコール	2.18%	Monoterpene alcohols	2.18%
リナロール	0.20%	Linalool	0.20%
α-テルピネオール	1.94%	α-Terpineol	1.94%
ゲラニオール	0.04%	Geraniol	0.04%
セスキテルペン炭化水素	0.13%	Sesquiterpene hydrocarbons	0.13%
β-カリオフィレン	0.13%	β-Caryophyllene	0.13%
セスキテルペンアルコール	0.04%	Sesquiterpene alcohols	0.04%
trans-ネロリドール	0.04%	trans-nerolidol	0.04%
エステル	0.24%	Ester	0.24%
酢酸オクチル	0.10%	octyl acetate	0.10%
酢酸リナリル	0.04%	linalyl acetate	0.04%
酢酸ゲラニル	0.10%	geranyl acetate	0.10%
	99.05%		99.05%

LO		*Lavandula angustifolia* P-98: PR
モノテルペン炭化水素	8.16%	Monoterpene Hydrocarbons
α-Pinene	0.14%	α-Pinene
α-ツジェン	0.05%	α-Tugen
カンフェン	0.23%	Camphene
β-ピネン	0.06%	β-Pinene
δ-3-カ レン	0.12%	δ-3-Carene
β-ミルセン＋α-フェランドレン	0.64%	β-Myrcene + α-Phellandrene
リモネン	0.28%	Limonene
β-フェランドレン	0.18%	β-Phellandrene
cis-β-オシメン	3.08%	cis-β-Osimene
γ-テルピネン	0.10%	γ-Terpinene
trans-β-オシメン	3.05%	trans-β-Osimene
p-サイメン	0.15%	p-Symene
テルピノレン	0.08%	Terpinolene
モノテルペンアルコール	40.91%	Monoterpene alcohols
trans-ツヤノール	0.06%	trans-tsuyanol
リナロール	36.34%	linalool
テルピネン-4-オール	2.25%	terpinen-4-ol
ラバンズロール	0.93%	lavansulol
α-テルピネオール	0.79%	alpha-terpineol
ネロール	0.16%	nerol
ゲラニオール	0.38%	geraniol
酸化物	1.36%	Oxide
1,8シネオール	0.50%	1,8-cineole
cis-リナロールオキシド	0.33%	cis-linalool oxide
trans-リナロールオキシド	0.21%	trans-linalool oxide
カリオフィレンエポキシド	0.32%	caryophyllene epoxide
セスキテルペン炭化水素	7.05%	Sesquiterpene hydrocarbons
α-サンタレン	0.62%	α-Santalene
cis-α-ベルガモテン	0.08%	cis-α-bergamotene
trans-α-ベルガモテン	0.14%	trans-α-bergamotene
β-カリオフィレン	3.58%	β-caryophyllene
β-ファルネセン	2.46%	β-farnesene
α-フムレン	0.10%	α-humulene
γ-カジネン	0.07%	γ-cadinene
セスキテルペンアルコール	0.05%	Sesquiterpene alcohol
trans-カジノール	0.05%	trans-cadinol
エステル	36.95%	Esters
酪酸ブチル	0.09%	Butyl butyrate
酢酸ヘキシル	0.35%	Hexyl acetate
酢酸1-オクテン-3-イル	0.74%	1-octen-3-yl acetate
酪酸ヘキシル	0.38%	Hexyl butyrate
酢酸リナリル	32.61%	Linalyl acetate
酢酸ラバンズリル	2.03%	Labanzuril acetate
酢酸ネリル	0.27%	Neryl acetate
酢酸ゲラニル	0.48%	Geranyl acetate
ケトン	1.73%	Ketone
3-オクタノン	1.15%	3-octanone
カンファー	0.49%	Camphor
クリプトン	0.09%	Crypton
脂肪酸アルコール	0.21%	Fatty alcohol
3-オクタノール	0.21%	3-octanol

NOTE: PRANAROM performs precise analysis by GC-MS chromatography on each new batch of essential oil in its laboratory to guarantee 100% pure chemotype quality. All officially imported PRANAROM essential oils are accompanied by a table of analysis of the ingredients necessary to confirm the quality of the oil. (The presented analysis table is a quality confirmation indicating that **the following items have been analyzed by an official laboratory registered with the Minister of Health, Labor and Welfare in Japan, and passed the acceptance criteria**). The table of ingredients shown here is an English translation of the table of ingredients attached to the essential oil used for the test.

Supplementary Data 2. The main components of mouse standard solid food.

Water	8.2	±	0.5	g
Crude Protein	23.3	±	0.4	g
Crude Fat	5.1	±	0.2	g
Crude Ash	5.8	±	0.2	g
Crude Fiber	3	±	0.2	g
Soluble nitrogenous material	54.6	±	0.6	g
Calories	357.3	±	2.5	Kcal
Vitamin A	1781	±	598	IU
Vitamin D3	130	±	31	IU
Vitamin E	8.9	±	0.6	mg
Vitamin B1	1.96	±	0.13	mg
Vitamin B2	0.99	±	0.05	mg
Vitamin C	5	±	1	mg
Calcium	1.02	±	0.04	g
Phosphorus	0.82	±	0.04	g
Magnesium	0.24	±	0.01	g
Sodium	0.20	±	0.01	g
Potassium	0.94	±	0.03	g
